# Predicting day-one mobility in partial nephrectomy patients using preoperative and intraoperative clinical parameters

**DOI:** 10.3389/fonc.2025.1528834

**Published:** 2025-05-16

**Authors:** Meijuan Xu, Qiuxuan Zhang, Xiaohui Mo, Yanmei Liu, Man Peng, Xuexia Ma

**Affiliations:** Department of Urology, Sun Yat-Sen Memorial Hospital, Sun Yat-Sen University, Guangzhou, Guangdong, China

**Keywords:** renal tumor, partial nephrectomy, postoperative day-one ambulation, enhanced recovery, predictive model

## Abstract

**Objective:**

To identify key factors influencing early postoperative ambulation in patients undergoing partial nephrectomy for renal tumors and to construct a predictive model for day-one ambulation based on these factors.

**Methods:**

This retrospective study analyzed 137 patients who underwent partial nephrectomy for renal tumors at the Department of Urology, Sun Yat-sen Memorial Hospital, between October 2020 and June 2023. Patients were randomly divided into a training set (n=97) and a test set (n=40) in a 7:3 ratio. Univariate and multivariate logistic regression analyses were conducted to evaluate potential risk factors influencing postoperative ambulation.

**Results:**

Of the 137 patients, 116 were able to ambulate on the first postoperative day. Significant factors associated with early postoperative ambulation included age, hypertension, tumor size, serum cystatin C, blood urea nitrogen, renal artery clamping time, and intraoperative blood loss. A predictive model was constructed based on age, tumor size, and intraoperative blood loss, demonstrating strong accuracy with areas under the receiver operating characteristic (ROC) curve of 0.902 in the training set and 0.975 in the test set. Bootstrap calibration curves confirmed the model’s predictive accuracy, and decision curve analysis (DCA) demonstrated a substantial clinical benefit.

**Conclusion:**

Age, tumor size, and intraoperative blood loss are key predictors of day-one ambulation in patients undergoing partial nephrectomy. This predictive model provides clinicians with a reliable tool for assessing early postoperative mobility, supporting enhanced recovery protocols and improving patient outcomes.

## Introduction

Renal cell carcinoma is among the most common urological cancers worldwide, and advancements in surgical techniques have significantly improved treatment outcomes and recovery processes for patients (1). Robotic-assisted partial nephrectomy has become the predominant surgical approach for treating localized renal tumors, largely replacing conventional laparoscopy due to its superior visualization, enhanced dexterity, and improved perioperative outcomes (2). Recent studies exploring novel robotic systems, such as the Hugo™ RAS platform, have further highlighted advances in surgical feasibility, perioperative outcomes, and trifecta achievement rates compared to conventional techniques (3, 4). These improvements in surgical techniques have intensified the focus on optimizing postoperative recovery protocols, including early ambulation strategies to enhance patient outcomes. As the focus on postoperative recovery grows, early mobilization has emerged as a key factor in enhancing patient outcomes, contributing to faster recovery times, lower complication rates, and improved long-term health trajectories.

Enhanced Recovery After Surgery (ERAS) protocols have shown substantial benefits across various surgical specialties, including urology, by reducing hospital stays, controlling costs, and improving patient satisfaction (5). For partial nephrectomy patients, early ambulation on postoperative day one (EA-POD1PN) is critical for minimizing complications, accelerating discharge, and reducing the burden on healthcare resources (6). Evidence suggests that early ambulation on the first postoperative day correlates with fewer complications, quicker recovery, and optimized hospital resource utilization (7). Despite its importance, there is a lack of reliable, evidence-based tools for predicting EA-POD1PN, which depends on a range of factors including preoperative health status, intraoperative variables, and early postoperative responses.

In recent years, clinical prediction models based on statistical methods such as logistic and Cox regression have been widely applied in medical fields including oncology and cardiovascular disease, where they help identify prognostic factors and support clinical decision-making (8). However, the prediction of postoperative outcomes for partial nephrectomy patients remains underexplored (9). Building on these advancements, our study aims to develop a predictive model for EA-POD1PN by leveraging a comprehensive dataset of preoperative, intraoperative, and postoperative factors. Through systematic analysis of these variables, we seek to create an interpretable model that can reliably predict early ambulation potential, supporting clinicians in crafting personalized rehabilitation strategies for optimal recovery.

We address a critical gap in the postoperative care of renal tumor patients by providing a predictive model that facilitates early risk identification and enables timely, targeted interventions. By enhancing our understanding of EA-POD1PN, we hope to deliver a practical tool with significant potential to improve postoperative outcomes for partial nephrectomy patients.

## Methods

### Study design and population

This retrospective study analyzed patients who underwent partial nephrectomy at Sun Yat-sen Memorial Hospital, Sun Yat-sen University, with the aim of developing a predictive model for EA-POD1PN. Patients were divided into a training set (N=97) and a testing set (N=40) to evaluate model performance. Baseline demographic, clinical, and surgical data were collected from electronic medical records.

### Data collection and variables

Collected variables included demographic information (e.g., age, BMI), comorbidities (e.g., hypertension, diabetes, heart disease), and laboratory parameters (e.g., WBC, LYM, NEU, Ccr, cystatin C). Tumor characteristics (e.g., tumor size) and intraoperative factors (e.g., surgery duration, blood loss, occlusion time) were also recorded. The primary outcome of interest was EA-POD1PN, defined as a patient’s ability to ambulate on the first postoperative day. Early ambulation was specifically defined as the ability to walk a minimum distance of 5 meters, with or without minimal assistance (e.g., walker or cane), as assessed by the ward physiotherapy team or attending nurse and documented in the electronic medical record. This operational definition is consistent with existing Enhanced Recovery After Surgery (ERAS) guidelines emphasizing early mobilization after surgery (10, 11).

### Model construction and validation

The predictive model was constructed using variables identified as significant predictors through univariable and multivariable logistic regression analyses in the training set. A nomogram was developed to estimate the probability of EA-POD1PN, incorporating the most predictive clinical factors identified. This scoring system provided an individualized risk estimate to support clinical decision-making.

For model validation, calibration was assessed by comparing predicted and observed probabilities of EA-POD1PN through calibration plots. Discrimination ability was evaluated by calculating the area under the receiver operating characteristic (ROC) curve (AUC) for both the training and testing sets (12), which reflects the model’s accuracy in distinguishing between patients who would and would not achieve EA-POD1PN.

### Clinical utility assessment

Decision curve analysis (DCA) was performed to evaluate the clinical utility of the model (13). By comparing net benefits of the predictive model against “treat-all” and “treat-none” strategies across a range of threshold probabilities, DCA helped determine the potential value of using the model in clinical practice, indicating its ability to provide benefit in real-world decision-making for postoperative ambulation management.

### Statistical analysis

All statistical analyses, data processing, and model training were conducted using R software version 4.3.1. A two-sided *P*-value < 0.05 was considered statistically significant, with 95% confidence intervals (CIs) calculated for each variable. Error bars represent 95% CIs, and all analyses adhered to the assumption checks for logistic regression, ensuring model reliability and interpretability.

## Results

### Baseline characteristics of the study cohort

A total of 137 patients who underwent partial nephrectomy were included in the study, divided into a training set (N=97) and a testing set (N=40). **Table 1** provides an overview of baseline characteristics, showing no statistically significant differences between the training and testing sets, thereby ensuring cohort comparability. The training and testing sets had similar distributions for key demographic and clinical variables.

**Table 1:** Baseline Data of Included Patients.

Variables	Training set (N=97)	Testing set(N=40)	P value
Sex			0.881
Male	54 (55.7%)	21 (52.5%)	
Female	43 (44.3%)	19 (47.5%)	
Age, year	49.000 [41.000, 60.000]	53.000 [35.750, 60.250]	0.960
BMI, kg/m^2^	23.423 [23.423, 23.423]	23.423 [23.423, 23.525]	0.126
Hypertension			0.276
Yes	30 (30.9%)	8 (20.0%)	
No	67 (69.1%)	32 (80.0%)	
Diabetes			
Yes	8 (8.2%)	2 (5.0%)	0.762
No	89 (91.8%)	38 (95.0%)	
Heart Disease			
Yes	1 (1.0%)	0 (0.0%)	1.000
No	96 (99.0%)	40 (100.0%)	
WBC	6.320 [5.190, 7.670]	6.455 [5.520, 7.280]	0.989
LYM	1.690 [1.450, 2.330]	1.720 [1.525, 2.148]	0.806
NEU	3.890 [2.930, 5.050]	4.140 [3.060, 4.867]	0.934
Ccr (umol/L)	76.000 [65.000, 91.000]	77.500 [67.000, 90.750]	0.769
Cystatin C	0.860 [0.720, 0.960]	0.863 [0.768, 0.903]	0.921
Urea nitrogen (mmol/L)	5.000 [4.100, 5.700]	5.000 [4.075, 5.825]	0.844
Tumor size (cm)	3.600 [2.700, 5.000]	4.090 [3.000, 4.825]	0.483
Surgery time (min)	115.000 [115.000, 115.000]	115.000 [108.750, 115.000]	0.040
Amount of bleeding (mL)	50.000 [20.000, 100.000]	50.000 [18.750, 100.000]	0.247
Occlusion time (min)	20.000 [17.000, 25.000]	20.000 [16.750, 21.500]	0.780
Surgery way			0.222
Robot-assisted laparoscopy	29 (29.9%)	17 (42.5%)	
Laparoscopy	68 (70.1%)	23 (57.5%)	
EA-POD1PN			1.000
Yes	82 (84.5%)	34 (85.0%)	
No	15 (15.5%)	6 (15.0%)	

LYM, Lymphocyte; NEU, Neutrophil; Cr, Creatinine.

### Univariable and multivariable analysis of predictive factors

Univariable logistic regression analysis (**Table 2**) identified several variables significantly associated with EA-POD1PN, including age, hypertension, cystatin C, urea nitrogen, tumor size, and intraoperative blood loss. In the multivariable analysis, age (OR=0.881, 95% CI: 0.806-0.942, *P*=0.001), tumor size (OR=0.611, 95% CI: 0.385-0.921, p=0.023), and blood loss (OR=0.992, 95% CI: 0.984-0.997, p=0.021) remained significant independent predictors of EA-POD1PN. These findings highlight the importance of these clinical factors in determining early ambulation potential and served as the foundation for the nomogram model.

**Table 2:** Univariable and multivariable analysis for patients with renal tumors who have undergone partial nephrectomy in training set.

Characteristics	Univariable analysis		Multivariable analysis	
	OR (95% CI)	P-Value	OR (95% CI)	P-Value
**Sex (Male *vs* Female)**	1.233 (0.407-3.977)	0.714		
**Age, year**	0.934 (0.887-0.977)	**0.005**	0.881 (0.806-0.942)	**0.001**
**BMI, kg/m^2^**	0.922 (0.615-1.340)	0.680		
**Hypertension (Yes *vs* No)**	0.321 (0.101-0.993)	**0.048**		
**Diabetes (Yes *vs* No)**	1.307 (0.208-25.410)	0.809		
**Heart Disease (Yes *vs* No)**	10662 (0-NA)	0.992		
**WBC**	0.960 (0.731-1.291)	0.777		
**LYM**	1.049 (0.453-2.618)	0.914		
**NEU**	0.980 (0.715-1.405)	0.904		
**Ccr (umol/L)**	0.989 (0.956-1.024)	0.518		
**Cystatin C**	0.007 (0-0.178)	**0.004**		
**Urea nitrogen (mmol/L)**	0.686 (0.471-0.977)	**0.038**		
**Tumor size (cm)**	0.745 (0.574-0.96)	**0.023**	0.611 (0.385-0.921)	**0.023**
**Surgery time (min)**	0.990 (0.970-1.012)	0.314		
**Amount of bleeding (mL)**	0.990 (0.982-0.996)	**0.002**	0.992 (0.984-0.997)	**0.021**
**Occlusion time (min)**	0.927 (0.849-1.010)	0.081		
**Surgery way (R-L *vs* L)**	3.191 (0.805-21.330)	0.144		

Bold values are statistically significant (p < 0.05). *OR* Odds ratio, *CI* confidence interval. LYM, Lymphocyte; NEU, Neutrophil; Cr, Creatinine; R-L, Robot-assisted laparoscopy; L, laparoscopy.

### Nomogram development and visualization

**Figure 1:**
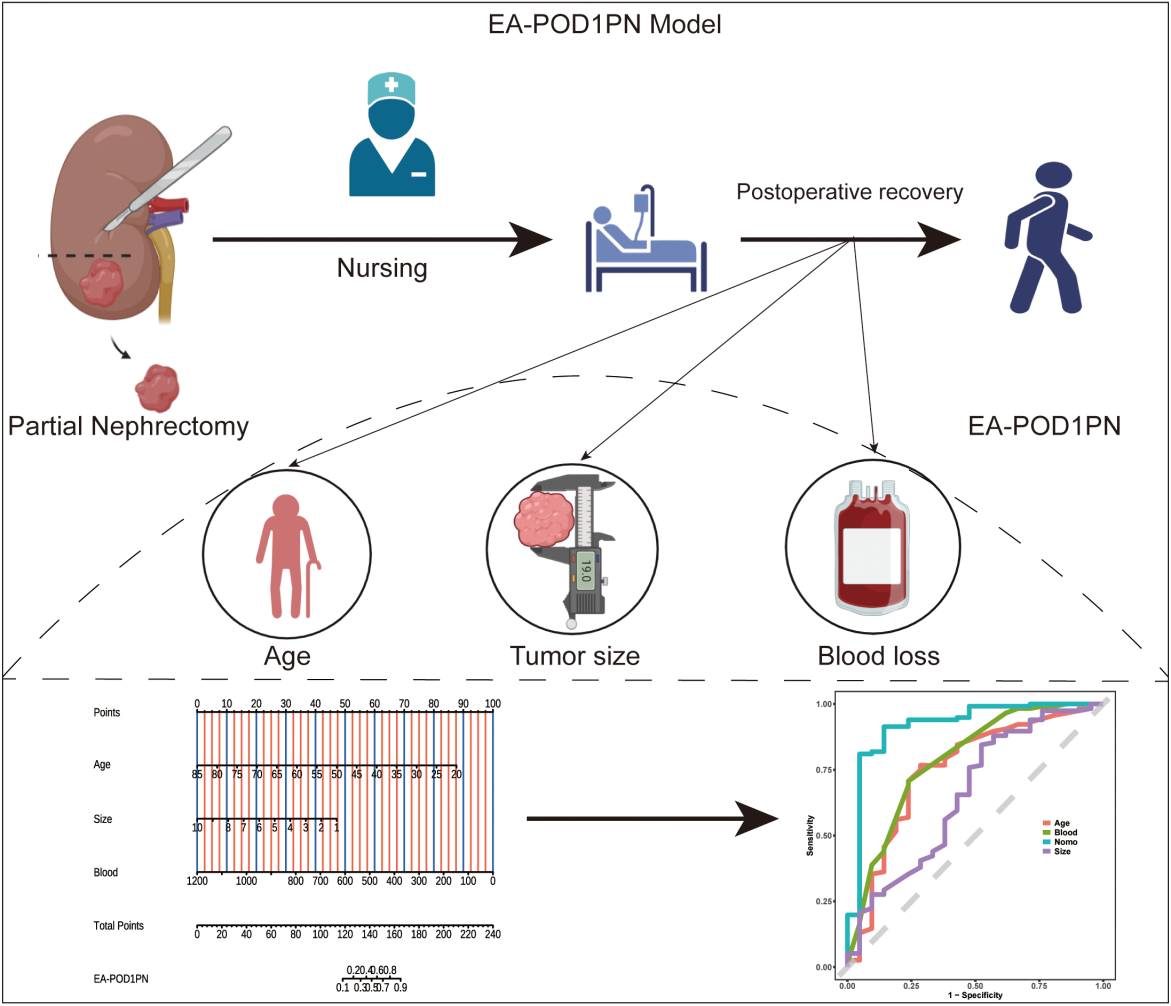
Schematic of the EA-POD1PN predictive model for patients undergoing partial nephrectomy.

**Figure 2:**
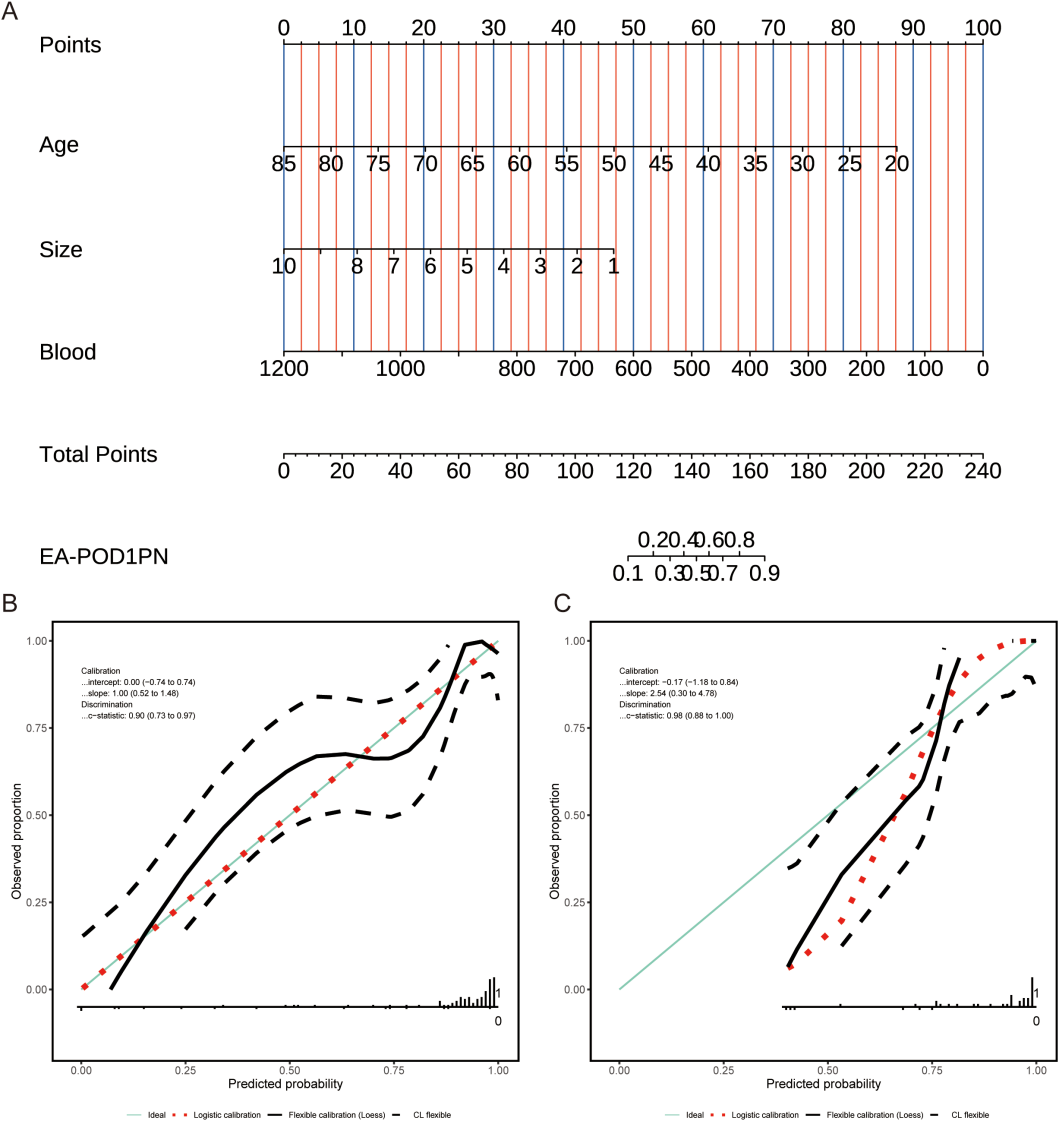
**(A)** Nomogram for predicting EA-POD1PN based on age, tumor size, and blood loss, with total points corresponding to the probability of early ambulation. **(B)** Calibration plot for the training set, showing agreement between predicted and observed probabilities. **(C)** Calibration plot for the testing set, indicating reliable performance of the model across datasets.

Based on the significant predictors identified in the multivariable analysis, a nomogram was developed to estimate the probability of EA-POD1PN (**Figure 1**). Each predictor—age, tumor size, and intraoperative blood loss—is assigned a specific point value, and the sum provides an individualized probability estimate for early ambulation. **Figure 2A** details the scoring system within the nomogram, illustrating how each variable contributes to the overall prediction.

### Calibration of the predictive model

The calibration of the nomogram was assessed by comparing predicted probabilities with observed outcomes in both the training and testing sets. Calibration plots (**Figures 2B, C**) demonstrated good agreement between predicted and observed probabilities, indicating that the model is well-calibrated and reliable across different cohorts.

### Model discrimination and predictive accuracy

**Figure 3:**
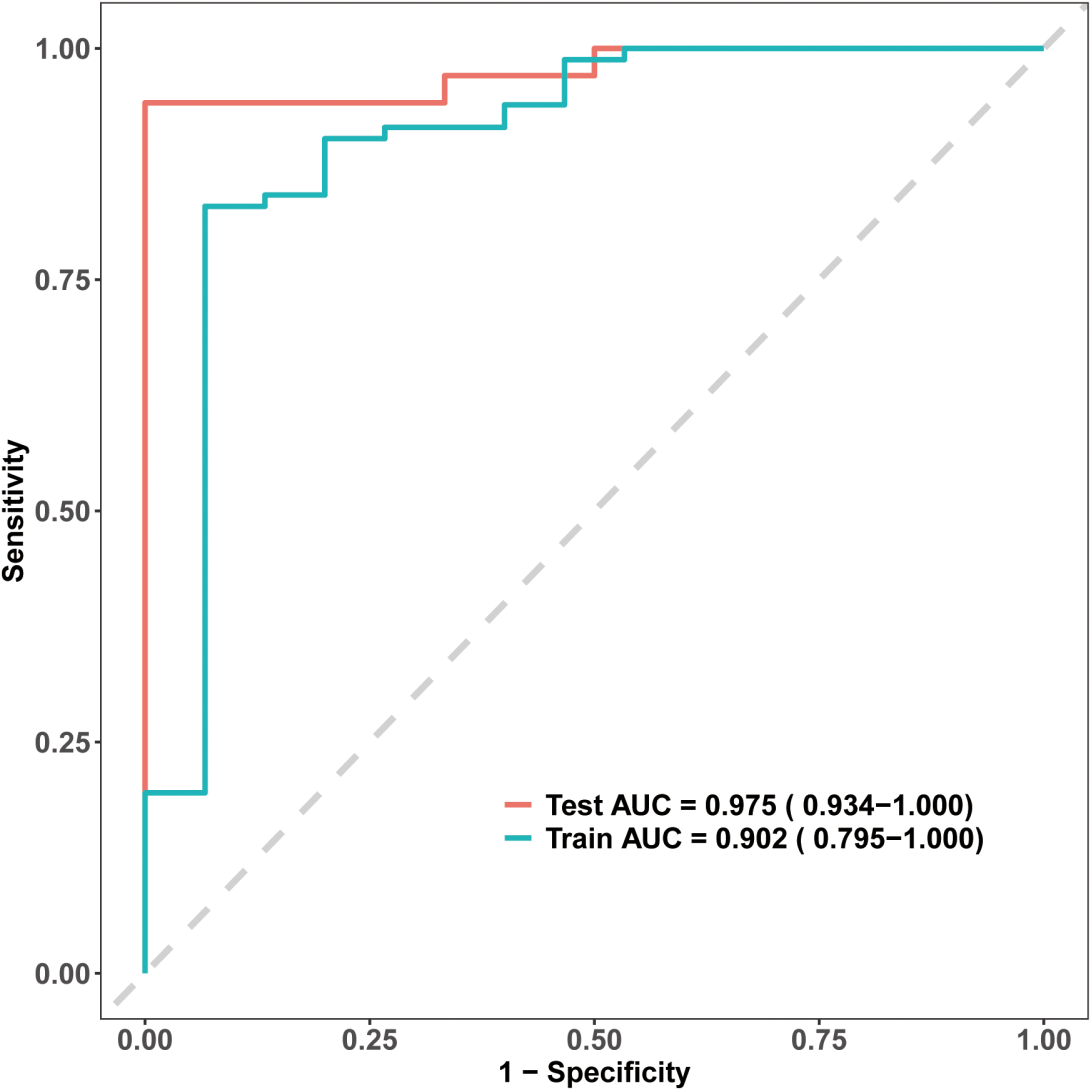
Receiver Operating Characteristic (ROC) curves for the EA-POD1PN predictive model. The model shows strong discriminative ability, with an AUC of 0.902 in the training set and 0.975 in the testing set, indicating high accuracy in predicting early ambulation.

The model’s discriminative ability was evaluated using receiver operating characteristic (ROC) curves. The nomogram demonstrated high discrimination, with an AUC of 0.902 in the training set and 0.975 in the testing set (**Figure 3**). These results underscore the model’s strong predictive performance and ability to effectively distinguish between patients likely and unlikely to achieve EA-POD1PN.

### Clinical utility through decision curve analysis

**Figure 4:**
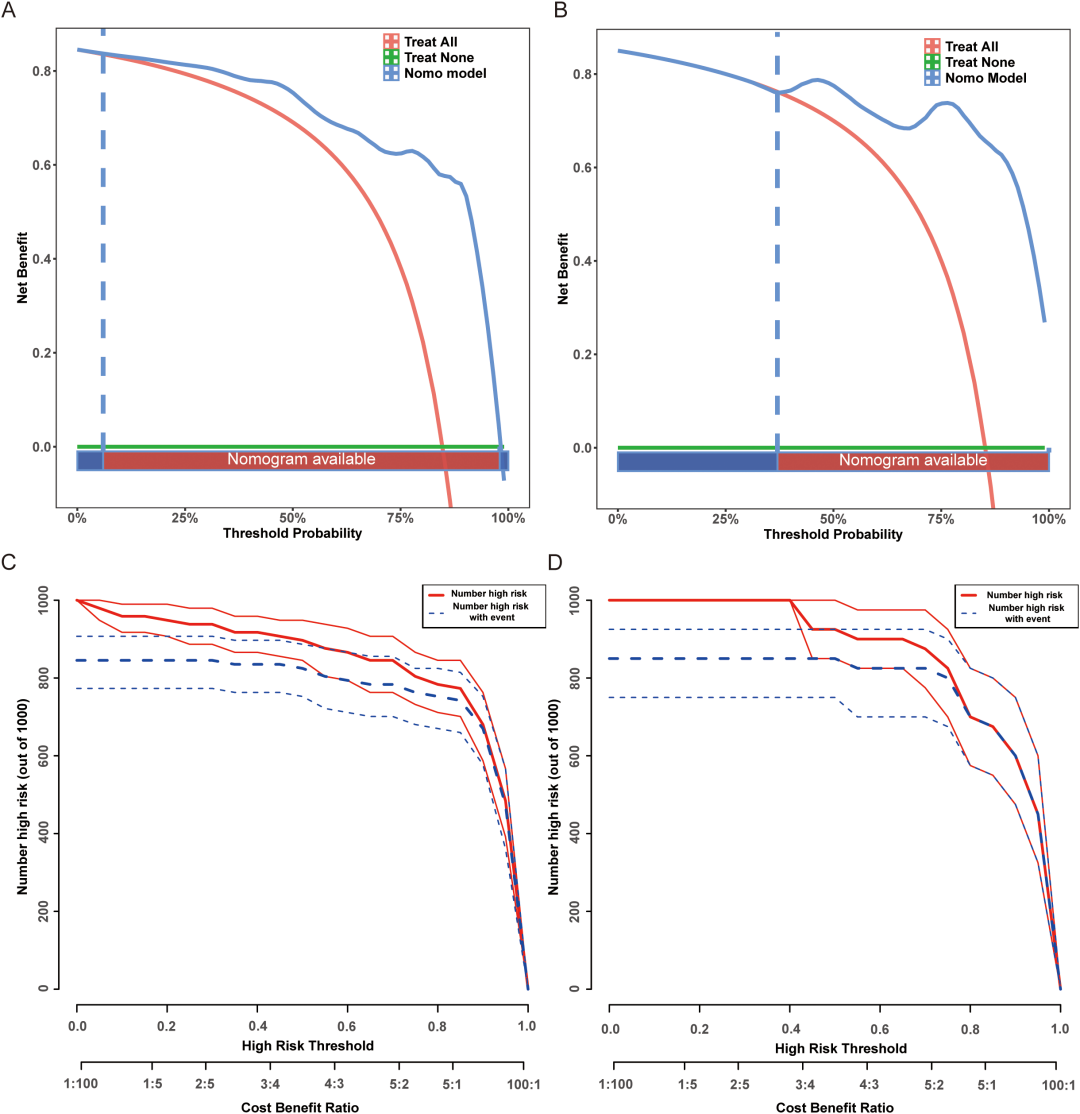
Decision curve analysis (DCA) and high-risk threshold analysis for the EA-POD1PN predictive model. **(A)** DCA for the training set, demonstrating net benefit across different threshold probabilities. **(B)** DCA for the testing set, confirming the model’s clinical utility. **(C)** High-risk threshold analysis for the training set, showing the number of patients classified as high risk at different thresholds. **(D)** High-risk threshold analysis for the testing set, supporting the model’s applicability in guiding postoperative interventions.

To evaluate the clinical utility of the EA-POD1PN nomogram, both decision curve analysis (DCA) and clinical impact curves (CIC) were generated. As shown in **Figures 4A, B**, the nomogram consistently provided a higher net benefit compared to the “treat-all” and “treat-none” strategies across a wide range of threshold probabilities, particularly between approximately 20% and 75%. This suggests that applying the model within this probability range can meaningfully inform postoperative management decisions. Additionally, the clinical impact curves (**Figures 4C, D**) illustrate the estimated number of patients identified as high risk for delayed ambulation at various threshold probabilities. These curves show that as the threshold increases, the number of predicted high-risk patients decreases, while the proportion of true positives remains relatively stable. This highlights the model’s potential for stratifying patients according to their risk and optimizing resource allocation for targeted interventions. Together, these findings underscore the practical utility of the nomogram in supporting individualized rehabilitation planning and enhancing early postoperative recovery in patients undergoing partial nephrectomy.

## Discussion

In this study, we developed and validated a predictive model for Early Ambulation on Postoperative Day One after Partial Nephrectomy (EA-POD1PN) by incorporating demographic, tumor-related, and intraoperative variables. Our model demonstrated strong predictive accuracy and discriminative ability, with AUC values of 0.902 in the training set and 0.975 in the testing set. This tool provides clinicians with a practical means to assess the likelihood of early ambulation in partial nephrectomy patients, potentially enhancing postoperative management and supporting individualized rehabilitation strategies tailored to each patient’s risk profile.

Our findings highlight age, tumor size, and intraoperative blood loss as significant predictors of EA-POD1PN. Older age was associated with a reduced likelihood of early ambulation, aligning with previous studies that identify age as a risk factor for delayed recovery and increased complications following major surgery (14, 15). This can be attributed to age-related declines in physiological reserve, impaired wound healing, reduced muscle strength, and a higher burden of comorbidities, all of which contribute to delayed postoperative mobilization. Tumor size and intraoperative blood loss, both markers of surgical complexity (16–18), were also inversely associated with EA-POD1PN. Larger tumors typically require more extensive dissection and longer operative times, resulting in greater tissue trauma, increased postoperative pain, and higher physiological stress, which may collectively hinder early ambulation. Similarly, greater intraoperative blood loss can lead to hypovolemia, anemia, and impaired tissue oxygenation, causing postoperative fatigue and hemodynamic instability, which negatively affect the patient’s ability to mobilize on the first postoperative day. These findings suggest that patients with larger tumors and higher blood loss may benefit from more intensive postoperative care and extended recovery protocols. This underscores the importance of preoperative planning and risk stratification, as identifying patients who may experience delayed recovery allows healthcare providers to anticipate potential challenges and implement preventive measures that can optimize outcomes.

The development of a nomogram based on these predictors facilitates a more personalized approach to postoperative risk assessment. By quantifying the risk of delayed ambulation, clinicians can identify high-risk patients who may benefit from targeted interventions such as enhanced pain management, early physiotherapy, or tailored rehabilitation protocols. This individualized approach aligns with the principles of Enhanced Recovery After Surgery (ERAS), which aim to optimize perioperative care, reduce complication rates, shorten hospital stays, and ultimately improve patient outcomes and healthcare efficiency (19). In this way, the nomogram supports not only predictive assessment but also the broader goals of patient-centered and resource-efficient care.

While prior research on partial nephrectomy outcomes has predominantly focused on surgical metrics like renal function preservation, blood loss, and complication rates, few studies have specifically examined early ambulation as an indicator of functional recovery (20–22). Moreover, our findings are consistent with results reported in other surgical disciplines. In enhanced recovery protocols for abdominal surgery, early mobilization has been shown to be significantly delayed in older patients and in those with more complex procedures or greater blood loss (5, 23). Similar observations have been made in thoracic surgery, where intraoperative factors such as prolonged operation time and intraoperative complications were associated with delayed ambulation (24). In orthopedic surgery, particularly after joint replacement, age, surgical invasiveness, and perioperative blood loss have also been highlighted as key predictors of delayed early mobilization (25). These parallels across surgical specialties support the generalizability of our model and underscore the fundamental role of patient physiological reserve and surgical burden in determining early postoperative mobility. Our study addresses this gap by focusing on early mobility, a critical determinant of both short-term recovery and longer-term quality of life. Functional recovery has a direct impact on patient well-being and satisfaction, influencing factors such as discharge readiness, risk of readmission, and overall rehabilitation trajectory (26, 27). Additionally, the model’s reliance on easily obtainable clinical variables enhances its feasibility and applicability in routine clinical settings, allowing for rapid, evidence-based assessments that can inform individualized care plans without requiring complex or costly tests.

The model’s robustness was confirmed through rigorous validation using calibration plots and decision curve analysis (DCA). Strong calibration across both training and testing cohorts demonstrates that the model accurately predicts observed outcomes, reinforcing its reliability for clinical application (28). Moreover, DCA results demonstrated that the nomogram provides substantial net benefit over a range of threshold probabilities between approximately 20% and 75%, which is clinically meaningful. Based on these findings, we propose that patients with predicted probabilities below 30% may benefit from prolonged monitoring and intensified rehabilitation interventions, while those with probabilities above 75% can follow standard postoperative pathways. This reinforces the model’s clinical utility, as it can assist healthcare providers in making informed decisions by identifying patients who are likely to benefit from targeted postoperative interventions, thus improving patient outcomes and optimizing resource allocation. The additional clinical impact curves further supported the model’s practical value in stratifying patients and guiding tailored postoperative management strategies.

Despite its strengths, this study has several limitations. First, the retrospective and single-center design may introduce selection bias, and external validation in larger, multicenter cohorts is necessary to confirm generalizability. Second, the relatively small sample size, especially in the testing set, may limit the statistical power and raise concerns of potential overfitting. Although we used a 7:3 train-test split for internal validation, we acknowledge that additional methods such as cross-validation or bootstrapping could further enhance model robustness. Third, the distribution of surgical approaches (robotic vs. laparoscopic partial nephrectomy) was unbalanced, reflecting an institutional transition toward robotic surgery during the study period; this heterogeneity may impact the generalizability of the model to centers performing exclusively robotic procedures. Future validation in purely robotic cohorts would strengthen the model’s applicability to contemporary surgical practice. Fourth, several potentially influential perioperative factors—such as anesthesia type, intraoperative complications, and postoperative pain management—were not included due to incomplete or unavailable records. Incorporating these variables in future studies may improve predictive accuracy. Lastly, this study focused solely on immediate postoperative mobility without assessing longer-term functional outcomes or quality of life. Further investigation into the relationship between early ambulation and broader recovery metrics would offer a more comprehensive evaluation of its clinical significance.

We aim to conduct prospective studies to validate our findings in larger and more diverse populations across various clinical settings. Additionally, developing advanced visualization and interpretation tools will be essential to improve model transparency and facilitate its use by clinicians in daily decision-making processes. Improved interpretability may encourage greater clinician confidence and engagement, fostering a more seamless integration of predictive models into clinical workflows. Ultimately, integrating predictive models like ours into routine postoperative care could support a proactive approach to patient management, enabling early identification of patients at risk for delayed recovery and enhancing the overall quality of postoperative care.

## Conclusion

Our predictive model for Early Ambulation on Postoperative Day One after Partial Nephrectomy (EA-POD1PN) offers a valuable tool for clinicians to assess early mobility potential in patients undergoing partial nephrectomy. By incorporating readily available clinical variables, this model has the potential to enhance postoperative management and support individualized rehabilitation strategies, ultimately improving patient outcomes. Future work will focus on expanding the model by integrating additional perioperative factors and validating its applicability across diverse clinical settings, thereby further refining its predictive accuracy and clinical utility.

## Data Availability

The raw data supporting the conclusions of this article will be made available by the authors, without undue reservation.
